# Negative life events and depression: a moderated mediation model of mobile phone addiction and chronotype among medical undergraduate students in North China

**DOI:** 10.3389/fpsyt.2026.1916367

**Published:** 2026-09-07

**Authors:** Yuxin Chen, Qiwei Wang, Yuhua Zhu, Siyu Li, Yueyang Hu, Zhengran Liu

**Affiliations:** 1 Department of Epidemiology, School of Public Health, Baotou Medical College, Baotou, China; 2 Inner Mongolia Student Bullying Prevention Research Center, Tongliao, China

**Keywords:** a moderated mediation model, chronotype, depression, mobile phone addiction, negative life events

## Abstract

**Objective:**

Although negative life events (NLEs) are known to be associated with depression, few studies have examined the potential mechanisms among medical undergraduates. In this exploratory study, we aimed to investigate relationships between NLEs and depression with the indirect effect of mobile phone addiction and the moderating effect of chronotype in a sample of medical undergraduate students in north China.

**Methods:**

This study was a cross-sectional survey using stratified random sampling method. A total of 1,616 students in a medical college in north China was randomly chosen. Participants were asked to complete four self-reported questionnaires on an online platform, including the Patient Health Questionnaire-9 (PHQ-9), the Morning and Evening Questionnaire-5 (MEQ-5), a self-designed scale about negative life events (NLEs) and mobile phone addiction.

**Results:**

This study indicated that, among the different dimensions of NLEs, study pressure, relationship setbacks and employment pressure significantly related to depression (*P* < 0.001). Furthermore, NLEs showed a significant indirect association with depression via mobile phone addiction. Additionally, chronotype significantly moderated the indirect effect (*P* < 0.001).

**Conclusions:**

Among medical undergraduates in north China, the association between NLEs and depression was indirectly associated with mobile phone addiction and moderated by chronotype, with a stronger indirect association observed in evening-type than morning-type individuals. Mobile phone addiction mediated and chronotype moderated the relationship between NLEs and depression. Morning chronotype can buffer depression with mobile phone addiction among Chinese medical undergraduate students, while evening chronotype can increase depression.

## 1 Introduction

Depression is a mental state characterized by lacking of energy, low mood, loss of interest, and diminished quality of life (1). The mental well-being of undergraduate students, particularly concerning depression, has garnered significant attention from both society and national policymakers. Globally, it was estimated that the prevalence of worldwide undergraduate students’ depression reached a high proportion of 25% (2). What’s worse, a meta-analysis revealed that medical undergraduate students had exhibited an even higher prevalence, with depression affecting 39.4% of this population (3). Therefore, research on depression should be conducted to explore mechanism, prevention and intervention of depression among medical undergraduate students.

Negative life events (NLEs) referred to adverse experiences perceived by individuals as distressing and unpleasant (4). NLEs constituted a well-established risk factor for depression across diverse age groups and ethnicities (5). Previous study has confirmed that stressful life events significantly increase the risk of depression among college students (6). Furthermore, college students encounter various types of NLEs, which have been shown to differentially impacts on depression. For example, in a study conducted at a Chinese university, academic pressure was an independent predictor of depression (*β* = 0.027, *P* < 0.001) (7). Concerning about post-graduation plans have also been reported as one of the three primary daily stressors among students at Franciscan University (8). Family-related issues represented another NLEs that was significant category of NLEs predictive of depression (9), particularly parental separation, whose adverse effects may persist over the long term (10). To date, studies have begun to explore the mechanisms underlying the association between NLEs and depression (11). Given the multiplicity of potential mediating pathways, further investigation is warranted to elucidate the complex mechanisms linking NLEs to depression. For example, Chen, G et al. proposed a mediation model suggesting that NLEs could indirectly affect subthreshold depression through coping styles, especially negative ones (12), while sleep quality could also be a mediator between NLEs and depression (13). What’s more, a longitudinal study confirmed perceived social support partially mediated the associations between them with a moderator of self-esteem among university freshmen (14). Given the multiplicity of potential mediating pathways, further investigation is warranted to elucidate the complex mechanisms linking NLEs to depression.

Mobile phone has become an indispensable part in our daily live. With the widespread adoption of mobile phones, negative consequences have emerged, including a series of addiction-like symptoms caused by excessive mobile phone use, which is referred to mobile phone addiction (15). Recent data indicated that in America, young people aged 18–29 had the highest mobile phone ownership rate at 97% across all age groups (16). Besides, among Chinese college students, the prevalence of mobile phone addiction was estimated at 36.35% (17), the proportion of severe using mobile phone had reached 25%, and the mobile phone addiction status had been rising from 2013 to 2022 with MPAI (The Mobile Phone Addiction Index) score increased by nearly 10 (18). In recent years, a number of studies have found the following relationships (especially in college students) that NLEs may result in mobile phone addiction (19, 20). While other studies have shown that mobile phone addiction significantly and positively predicted depression (21, 22). Based on the above empirical evidence, we can reasonably infer that mobile phone addiction may serve as a bridge between NLEs and depression.

This study used compensatory internet use theory (CIUT) (23) as the theoretical framework for understanding the effect of mobile phone addiction between NLEs and depression. According to CIUT, excessive phone use is not a pathological condition, but rather a maladaptive coping strategy employed by individuals in managing NLEs. Specifically, NLEs trigger negative emotions, which in turn motivate individuals to seek relief in the virtual world, and the habit may lead to internet addiction. Likewise, as a device to access the internet, a mobile phone could serve as an external tool for negative emotion regulation, and excessive dependency leads to mobile phone addiction. Therefore, we propose Hypothesis 1: mobile phone addiction mediates the relationship between NLEs and depression among Chinese medical college students.

However, individuals with the same level of mobile phone addiction may have different depression degrees. Some factors may have worked alleviating or increasing mobile phone addiction in developing into depression. For example, preference for solitude and neurotic personality could be moderators in their relationship (21, 24). Besides, some studies have incorporated sleep-related factors (such as sleep duration and sleep quality) into the internal mechanism linking NLEs and depression (25–27), but few about chronotype, another sleep-related factor, which may also play an important moderating role in that relationship.

Chronotypes refers to individual’s expression of different endogenous circadian rhythmicity patterns (28), which can be categorized to three typical types: morning type, intermediate type, and evening type (29). In other words, chronotype refers to a person’s preferred time schedule for daily routines, including time of waking and bedtime (30). A study targeting Chinese college students examined the effect of PMPU (Problematic Mobile Phone Use) trajectories on chronotype, whose conclusion was that a high-level score trajectory of PMPU may be a potential risk factor for evening chronotype among college students, and gender differences exist within this relationship (31). Regarding the relationship between chronotype and depression, a review indicates that morning chronotype may help alleviating depression, while evening chronotype may be a risk factor (32). Moreover, the association exhibits non-linear characteristics and symptom specificity (33). The concept of social jetlag (34) provides a crucial theoretical link. Social jetlag refers to the chronic misalignment between an individual’s biological clock and socially imposed schedules. For evening-type individuals, early school or work start times create a recurring mismatch between their internal circadian phase and external demands. This discordance—reflected in systematic shifts in sleep timing between weekdays and weekends—has been confirmed as a prevalent phenomenon among student populations worldwide (35). Recently a study have demonstrated that high social jetlag (≥1.2 hours) was associated with significantly increase in depression risk in Chinese college students and the association was non-liner (36). Therefore, we propose Hypothesis 2: chronotype would play a moderating role between mobile phone addiction and depression among Chinese medical college students.

**Figure 1:**
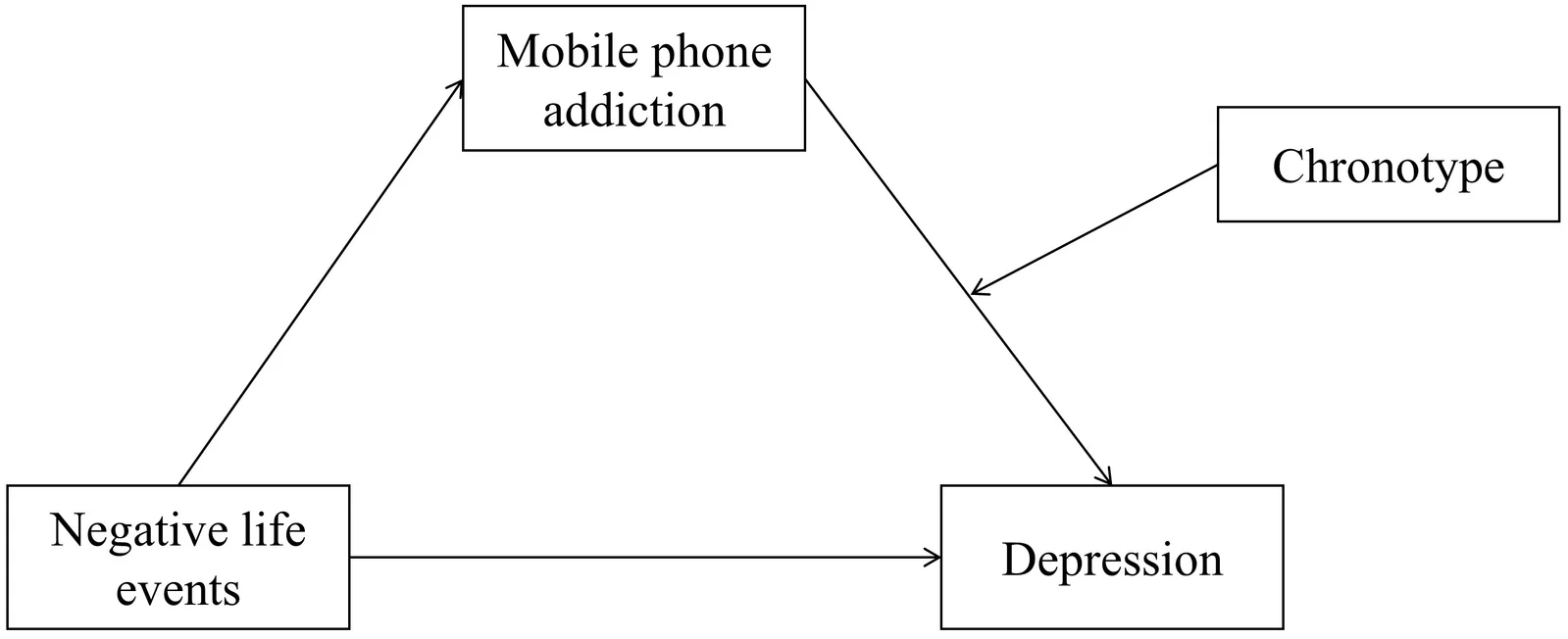
The conceptual framework of the moderated mediation model.

To sum up, this study proposed a moderated mediation model in the relationship between NLEs and depression with the indirect effect of mobile phone addiction and moderating effect of chronotype. (See **Figure 1**). To our knowledge, there are few systemic study to incorporate chronotype as a moderating factor in this mechanism among medical undergraduate students. Through this study, a new perspective would be provided to prevent and intervene depression based on individual’s chronotype among college students.

## 2 Materials and methods

### 2.1 Participants

This study was a cross-sectional survey conducted in October, 2025. A stratified random sampling method was used to select the study population. Firstly, a medical college in northern China was chosen as the study setting. Then, to ensure representativeness across different academic disciplines, equal number of students were randomly selected from four major departments (Public Health, Clinical Medicine, Medical Technology & Anesthesiology, and Stomatology). Specifically, considering the non-response rate of 20%, 440 students were randomly selected from each department. Finally, questionnaires were administered via an online survey platform. A total of 1706 participants responded to the survey, yielding a participation rate of 96.9%. For the present study, participants with missing data on key variables including negative life events, mobile phone addiction, chronotype, depression and demographics characteristics were excluded from the sample (n=90). Consequently, the final analytical sample comprised 1,616 participants.

The sample size was determined based on *a priori* calculations according to a meta-review of Gao et al., in which the prevalence of depression among Chinese university students is 28.4% (37). The following formula was used *n=Z^2^P(1−P)/d^2^*, where *n* is the sample size, the *Z* value (corresponding to the significance level p 0.05) is 1.96. P value is the expected occurrence of depression, and the *d* value is 0.1*P* (recommended value for an expected prevalence between 10 and 90% (38)). What’s more, questionnaire non-response rate (20%) should be considered. Based on the following calculation, the minimum sample size was 1,162. Our final analyzed sample size of 1,616 far exceeded these minimum requirements, providing over 95% statistical power to detect indirect effect while minimizing Type II errors.

### 2.2 Procedure

The study was approved by Institutional Review Board of Anhui Medical University and was conducted in accordance with the protocol of the Declaration of Helsinki (Ethical approval number: 81250724). This study emphasized that students were voluntary and anonymous, and given the right to withdraw from the survey at any point, even after it had started. After giving a briefing about study purpose and procedures, consents were obtained from every participant. An online platform called REDCap was used to distribute the questionnaire via a specific QR code. After scan the QRcode, the informed consent containing the above content will appear first. Only after checking the option of “I have read and agree the following content” could participants proceed to fill out the questionnaire, or they would be prompted to exit. All participants received information regarding accessing mental health support if they experienced any emotional distress during or after the study. Before the formal survey, the researchers conducted a pre-survey to ensure level of understanding of the participants.

### 2.3 Measures

The assessment of negative life events was assessed using a brief self-reported 4-item scale to enhance participant compliance, completion rates, and data accuracy. The scale measured the occurrence and emotional impact of four major negative life events among medical college students in the past 6 months, including family adverse events (e. g., parental divorce, serious illness or death of family members), study pressure (e. g., academic failure or exam-related pressure), relationship setbacks and future employment pressure. Each item was answered on a 4-point scale (1 = no event, 2 = minor impact, 3 = moderate impact, 4 = severe impact). Elevated total scores indicate heightened exposure to adverse life events and are associated with increased psychological distress. In the current sample, the instrument demonstrated good reliability, with the Cronbach’ s alpha of this scale was 0.733, which is above the conventional threshold of 0.70. As emphasized by Taber (39) and Schmitt (40), forced homogeneity is neither expected nor desirable for instruments measuring diverse life stressors. Thus, given that negative life events were intrinsically heterogeneous across different life spheres, making an alpha of 0.733 highly satisfactory for establishing measurement reliability. Thus, it offered a concise yet accurate assessment of exposure and psychological impact.

Depression was evaluated utilizing the Patient Health Questionnaire-9 (PHQ-9) (41), a widely validated instrument for assessing the severity of depression. This self-report measure comprises nine items. Each item was rated on a 4-point scale from 0 = not at all to 3 = nearly every day, yielding a total score ranging from 0 to 27. Higher scores indicated more severe of depression. Depression was defined as a score greater than or equal to 5 (41). It has been confirmed that PHQ-9 has great validity and reliability in Chinese college students (42). In this study, the Cronbach’ s alpha of the scale was 0.902 and construct validity was acceptable.

Mobile phone addiction was measured by a self-developed 13-item scale. Participants rated each item on a 5-point Likert scale (1 = completely disagree to 5 = completely agree). High scores indicated higher levels of mobile phone addition. In this study, the Cronbach’s alpha of the scale was 0.925 and construct validity was acceptable.

Chronotype was accessed by using the Morning and Evening Questionnaire-5 (MEQ-5) (43), an abbreviated 5-item adaptation of the original 19-item MEQ designed for efficient classification of individual chronotypes (morning type, intermediate type, or night type). This instrument evaluates key chronobiological parameters including sleep-wake preferences, morning alertness levels, optimal performance windows, and subjective self-evaluation. The composite score ranged from 4 to 25. Lower scores indicated closer to night type, while higher scores suggested closer to morning type. The Chinese version used in this study have been verified previously (44). In this study, the Cronbach’ s alpha of the scale was 0.866 and construct validity was acceptable.

Covariates mainly about demographic information were taken into account in this study, including gender, grade, nation, one-child status, residence, BMI and parental educational level. Criteria of weight for adults are employed to categorize participants as underweight (BMI<18.5 kg/m^2^), normal weight (18.5≤BMI<24.0 kg/m^2^), overweight (24.0≤BMI<28.0 kg/m^2^) and obesity (BMI ≥28.0 kg/m^2^) (45).

### 2.4 Statistical analyses

Statistical analyses were conducted using IBM SPSS Statistics, Version 26.0 (IBM Corp., Armonk, NY, USA). The differences of the score of depression in different demographic characteristics were examined by t-test and one-way analysis of variance (ANOVA). The predictive effects of distinct dimensions of negative life events on depression were analyzed using multiple liner regression. Pearson correlation was used to evaluate the bivariate relationships among negative life events, depression, mobile phone addiction and chronotype. The mediation model (Model 4) and the moderated mediation model (Model 14) were tested using the PROCESS macro (46). The indirect effects were tested with bias-corrected bootstrapping (n = 5000) and 95% confidence intervals (*CI*) for the indices. It indicated the statistically significant of the parameter if a 95% bootstrapped *CI* exclude zero.

## 3 Results

The demographics of subjects and their effects on depression were displayed in **Table 1**. The prevalence of depression in the overall sample was 33.9%, and moderate or higher depression was 6.8%. The final sample (n=1,616) comprised 66.3% female and 33.7% male. Univariate analysis indicated that gender (*P* < 0.05) and grade (*P* < 0.001) were associated with depression.

**Table 1:** Demographics of subjects and their effect on depression(n=1,616).

Variables	Groups	n	*Mean*	*SD*	*t/F*	*P*
Gender	Meal	544	3.29	4.421	-2.364	0.018
	Female	1072	3.82	4.168		
Grade	Freshman	530	3.15^ab^	3.767	7.861	<0.001
	Sophomore	387	4.49^bc^	4.970		
	Junior	397	3.66^cd^	4.252		
	Senior	302	3.41^bd^	3.953		
Nation	Han nationality	1268	3.58	4.220	-1.222	0.222
	Other nationalities	348	3.89	4.405		
One-child status	Yes	662	3.59	4.214	-0.416	0.678
	No	954	3.68	4.295		
Residence	Central areas of large and medium-sized cities	342	3.38^ab^	4.280	2.327	0.054
	urban suburbs	167	4.52^bc^	4.833		
	County-level city or county-level municipality	565	3.55	4.190		
	Township	210	3.46^bd^	4.168		
	Rural areas	332	3.75	4.076		
BMI	Underweight	217	3.65	4.660	0.152	0.928
	Normal weight	939	3.59	4.165		
	Overweight	291	3.70	4.025		
	Obesity	169	3.82	4.666		
Father’s educational level	Primary school and below	254	3.48	3.768	1.074	0.368
	Junior high school	651	3.67	4.282		
	Senior high/technical secondary school	379	3.97	4.411		
	Junior college/bachelor	300	3.37	4.245		
	Postgraduate and above	32	3.19	5.659		
Mother’s educational level	Primary school and below	350	3.83^ad^	4.239	2.092	0.080
	Junior high school	591	3.60	4.206		
	Senior high/technical secondary school	360	3.99^cd^	4.485		
	Junior college/bachelor	286	3.07	3.889		
	Postgraduate and above	29	3.55	5.773		

a, b, c, d indicate statistically significant differences between groups, *post hoc* analyses were used by LSD (Least Significant Difference).

The associations between different dimensions of negative life events and depression were showed in **Table 2**. Among the four dimensions, study pressure (*β* = 0.267, *P* < 0.001), relationship setbacks (*β* = 0.194, *P* < 0.001) and employment pressure (*β* = 0.288, *P* < 0.001) were significantly associated with high levels of depression.

**Table 2:** Associations between dimensions of negative life events and depression.

Independent variables	*β*	*t*	*P*	*95%CI*
Family adverse events	0.029	1.345	0.179	[-0.100,0.536]
Study pressure	0.267	11.364	<0.001	[1.095,1.551]
relationship setbacks	0.194	8.800	<0.001	[1.017,1.601]
employment pressure	0.288	11.905	<0.001	[1.081,1.508]

Adjusted for gender, grade, nation, one-child status, residence, BMI, father’s educational level and mother’s educational level.

Descriptive statistics and correlation analysis were conducted for negative life events, mobile phone addiction, depression, and chronotype. Negative life events were positively correlated with both mobile phone addiction (*r* = 0.293, *P* < 0.001) and depression (*r* = 0.558, *P* < 0.001). In addition, negative life events (*r* = -0.142, *P* < 0.001), mobile phone addiction (*r* = -0.170, *P* < 0.001) and depression (*r* = -0.221, *P* < 0.001) were all negatively correlated with chronotype. All four dimensions of negative life events showed positively associated with mobile phone addiction and depression, while negatively associated with chronotype except family adverse event (See in **Table 3**).

**Table 3:** Descriptive statistics and correlations among variables.

Variables	*M*	*SD*	1	2	3	4	5	6	7	8
1.Negative life events	5.577	2.069	1							
2.Family adverse events	1.161	0.565	0.531^***^	1						
3.Study pressure	1.477	0.860	0.747^***^	0.200^***^	1					
4.Relationship setbacks	1.213	0.633	0.592^***^	0.252^***^	0.203^***^	1				
5.Employment pressure	1.726	0.949	0.800^***^	0.214^***^	0.473^***^	0.295^***^	1			
6.Mobile phone addiction	22.113	8.564	0.293^***^	0.092^***^	0.221^***^	0.197^***^	0.255^***^	1		
7.Depression	3.644	4.261	0.558***	0.192***	0.447***	0.339***	0.475***	0.416***	1	
8.Chronotype	14.653	3.264	-0.142^***^	-0.005	-0.120^***^	-0.084^**^	-0.144^***^	-0.170^***^	-0.211^***^	1

^**^P < 0.01, ^***^P < 0.001. Adjusted for gender, grade, nation, one-child status, residence, BMI, father’s educational level and mother’s educational level.

As shown in **Table 4**, the association between negative life events and depression was positively significant (*β* = 1.149, *P* < 0.001). It showed the significantly indirect association between negative life events and depression through mobile phone addiction. The indirect effect accounted for 14.53% of the total relation between NLEs and depression. We calculated 95% confident intervals (*CI*) based on 5,000 bootstrap resampling. The indirect association between negative life events and depression via mobile phone addiction (95% *CI*= [0.115,0.231]) were significant, as zero was excluded in the 95% *CI*.

**Table 4:** Mediation analysis of negative life events on depression.

Independent variables	Model 1 (depression)		Model 2 (mobile phone addiction)		Model 3 (depression)	
	*β*	*t*	*β*	*t*	*β*	*t*
Negative life events	1.149	26.923^***^	1.217	12.279^***^	0.982	23.204^***^
Mobile phone addiction					0.137	13.462^***^
*R2*	0.316		0.088		0.385	
*F*	82.438^***^		17.151^***^		100.644^***^	
Model effect	Effect		SE		t	
*Total effect*	1.149		0.043		26.923^***^	
*Direct effect*	0.982		0.042		23.204^***^	
*Indirect effect*	0.167		0.029		95%*CI* [0.115,0.231]	

^***^P<0.001. Adjusted for gender, grade, nation, one-child status, residence, BMI, father’s educational level and mother’s educational level.

**Figure 2:**
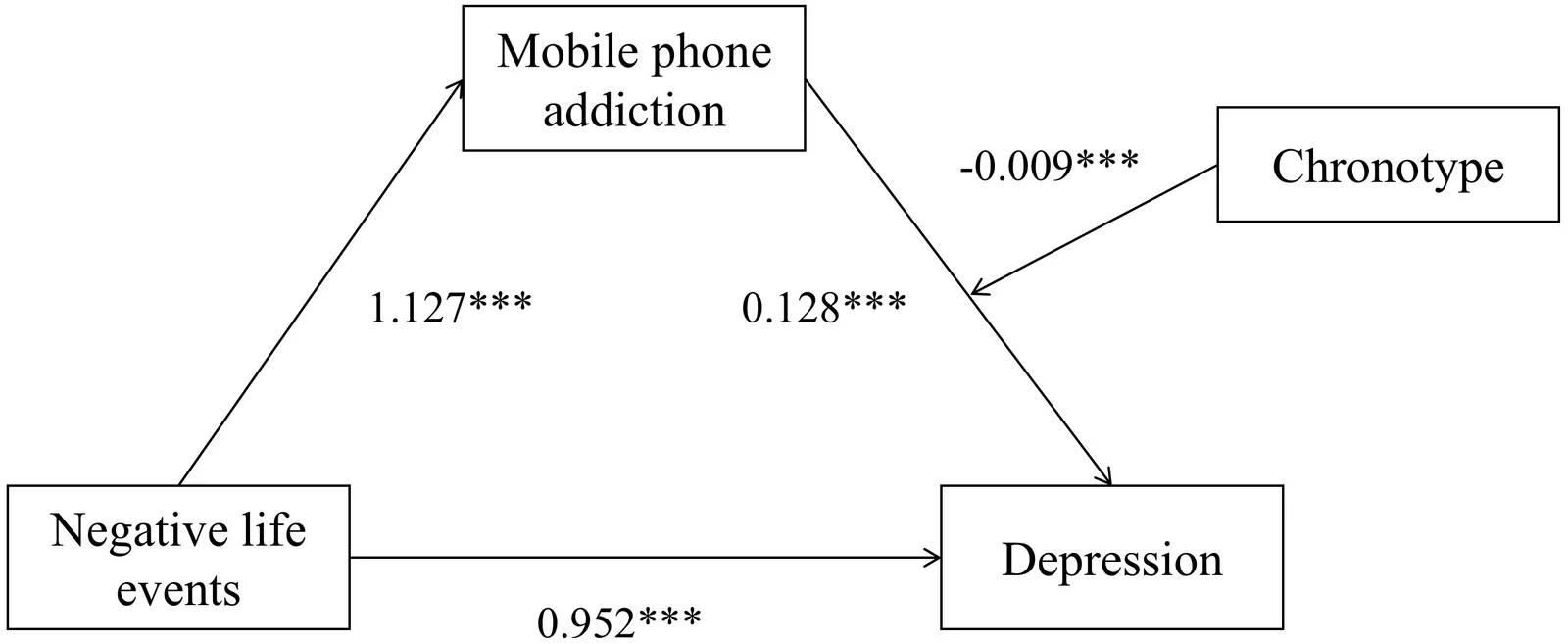
Moderated mediation model of negative life events on depression. ***P<0.001. Adjusted for gender, grade, nation, one-child status, residence, BMI, father’s educational level and mother’s educational level.

The results of the moderated mediation model were presented in **Table 5** and **Figure 2**. First, the Model 14 test in the application process of this study tested the theoretical hypothesis model. Model 3 was significant (*F* = 89.006, *P* < 0.001, *R^2^* = 0.400). The interaction term between mobile phone addiction and chronotype was related to depression, indicating that chronotype moderated the relationships between the mobile phone addiction and depression.

**Table 5:** Testing the moderated mediation effect of negative life events on depression.

Independent variables	Model 1 (depression)			Model 2 (mobile phone addiction)			Model 3 (depression)		
	*β*	*se*	*t*	*β*	*se*	*t*	*β*	*se*	*t*
Negative life events	1.149	0.043	26.923^***^	1.127	0.099	12.279^***^	0.952	0.042	22.582^***^
Mobile phone addiction							0.128	0.010	12.614^***^
Chronotype							-0.128	0.026	-4.942^***^
Mobile phone addiction *Chronotype							-0.009	0.002	-3.626^***^
*R^2^*	0.316			0.088			0.400		
*F*	82.438^***^			17.151^***^			89.006^***^		

^***^P<0.001. Adjusted for gender, grade, nation, one-child status, residence, BMI, father’s educational level and mother’s educational level.

**Figure 3:**
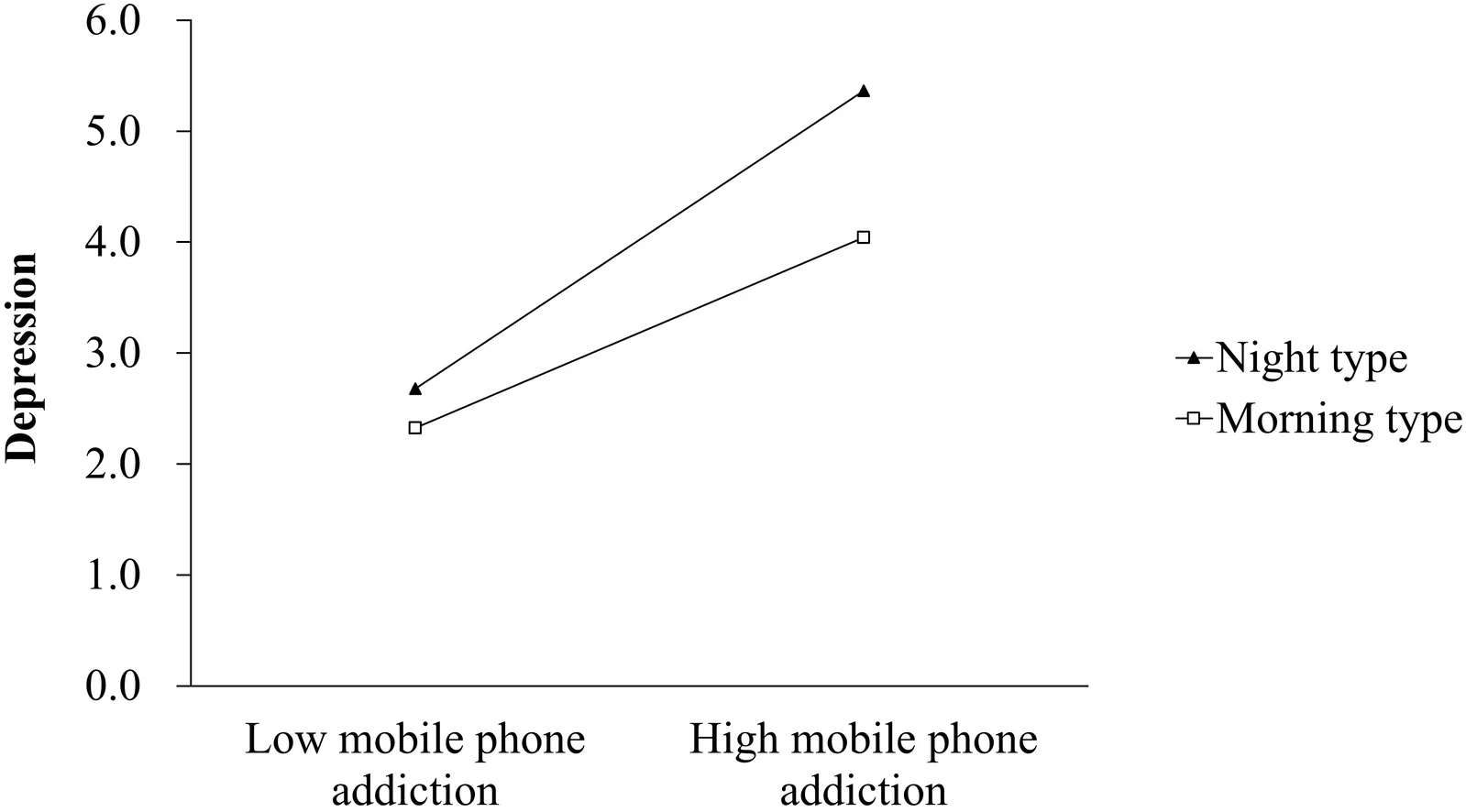
Chronotype moderates the effect of mobile phone addiction on depression.

The severity of depression also rose with an increase in the degree of mobile phone addiction. **Figure 3** demonstrated a steeper slope of the association between mobile phone addiction and depression among night-type individuals than morning-type individuals (*β_night-type_*= 0.191, *β_morning-type_*= 0.122). **Table 6** further corroborated this finding by showing a significant difference in a pairwise contrast of the conditional indirect effects (*95% CI* = [-0.127, -0.010]). They collectively demonstrated that mobile phone addiction was more strongly related to depression at night type compared to morning type.

**Table 6:** Conditional indirect effect of negative life events on depression.

Level of chronotype	Effect	*BootSE*	*95% CI*
1. Night type (*M-1SD)*	0.191	0.035	[0.127,0.267]
2. Morning type (*M+1SD)*	0.122	0.023	[0.083,0.174]
Pairwise contrast (effect 2 minus effect 1)	-0.069	0.029	[-0.127, -0.010]

^***^P<0.001. Adjusted for gender, grade, nation, one-child status, residence, BMI, father’s educational level and mother’s educational level.

## 4 Discussion

This study analyzed the indirect role of mobile phone addiction between negative life events (NLEs) and depression. Furthermore, the study also determined whether chronotype moderate the aforementioned effect. The moderated mediation model provides empirical evidence among Chinese medical college students.

Compared with prior studies (47), among Chinese college students, the detection rate of depressive symptoms in our study is higher, which indicates that medical students may possess a worse mental health status than general undergraduate students. This may attribute to the training plan of medical college. Students face intensive nature of theoretical courses as well as conduct internship at hospital with a generally longer duration of study compared with other disciplines (48). What’s more, due to the annually rising enrollment numbers of medical colleges while the recruiters’ academic requirements for medical institutions are gradually increasing, medical college students are facing immense employment pressure (49). These reasons may all contribute to the depression detection status in this study. However, the prevalence of depression is milder compared with a study during COVID-19 (50), which indicates that students may be gradually recovering from the trauma caused by the pandemic. What’s more, according to our results, all grades were associated with depression, in which sophomore achieved the highest average scores of 4.49 (*SD* 4.970). Despite the use of different depression assessment scales, the same phenomenon was also observed in a study conducted among college students (51), which may be attribute to heavier academic workload, coupled with more practical course requirements and participation in extracurricular activities in sophomore.

### 4.1 Association of NLEs’ dimensions with depression

Another interesting finding is that NLEs across different dimensions have significantly different impact on depression. Firstly, people with more NLEs are more addicted to depression. Although differed in subjects, previous research found that negative life events across multiple dimensions could lead to depression, which consisted with our results (52–54). As mentioned before, due to the complexity of theoretical and practical aspects in medical education and the intensity of employment competition, medical college students are usually facing more pressure from various aspects, especially in study, relationship and employment. These pressures reduce students’ ability to cope with stress (55). Consequently, the negative impacts of stress are amplified, leading to a decline in mental health status, including the onset or exacerbation of depressive symptoms (56).

Further analysis revealed that pressure from employment and study has greater impact on depression than relationship, which is consistent with previous research findings (57). On one hand, according to Higgins’ self-difference theory, the discrepancy between the actual self and the ideal self leads to a lack of positive outcomes, which is associated with depressive emotions (58). In this study, high-intensity academic and occupational stress represents a concentrated manifestation of this self-difference and thus exhibits a strong predictive effect on depression. On the other hand, college students have open social networks. Although relationship setbacks may trigger interpersonal trauma and lead to depression (59), they can compensate by making new friends or turning to virtual social interactions, thereby mitigating the direct impact on depression. Moreover, our study showed that adverse family factors may not the predictor of depression. However, Maajeeny, H have proved that children who had experienced parents’ divorce were more likely to have depression (60), which inconsistent with our results. One reason maybe that the life environment of college student are usually more school than family, so the passive impact of family adverse events on depression can be impaired due to not closely connected with family. Another reason, current research typically describes family negative events as a state (e.g., parental marital status) rather than recently experienced events. So in our study, the occurrence frequency of family adverse events in six months may be relatively low, which may limit the statistical significance of this variable.

### 4.2 Indirect role of mobile phone addiction

The results from our study support the hypothesis that mobile phone addiction served as a partial indirect pathway in the association between NLEs and depression. The results indicated that the negative effects of NLEs on depression can be partially explained by mobile phone addiction, which lined with previous studies (56).

Pressure in study and life lead individuals to seek alternative forms of satisfaction in the virtual world (usually using mobile phone) to alleviate their tendency toward boredom (20, 61). Having been in a prolonged poor adaptation of NLEs can lead to excessive mobile phone usage and forms mobile phone addiction, which support compensatory internet use theory (CIUT) mentioned before. According to a meta-analysis, individuals with excessive mobile phone use have activation of several brain areas that responsible for voluntary and automatic emotion control (62). Therefore, it was tempting to speculate that, when facing with NLEs, individuals may use mobile internet to escape from reality, leading to mobile phone addiction. If things continue this way, mobile phone addiction can result in impaired emotional regulation, ultimately increase the risk of depression occurrence.

However, our study has a smaller proportion of indirect effect compared to the existing literature (14.53% to 29.06%) (56). Maybe the difference in the professional backgrounds of the participants across both studies is one contributing factor. Besides, according to Hafeman (63), the prevalence of NLEs, the potential confounders, even the direct effect can affect the indirect effect. Therefore, the discrepancy in the proportions of indirect effects between the two studies can be attributed to differences in the measurement methods used for independent variables, the inclusion of varying confounding factors, distinct sample sources and so on.

### 4.3 Moderator role of chronotype

In addition, our research indicated that mobile phone addiction can increase the risk of depression, while chronotype can moderate that relationship. To be specific, evening type could amplify this impact of mobile phone addiction on depression while morning type could mitigate this impact. Chronotype is the inter-individual differences of biological clocks’ entrainment to light-dark cycles (64). It means that chronotype reflects individuals’ preference of time schedule, which is determined by genetic variance and circumstance. Morning chronotype usually aligns with social rhythms, while evening chronotype often has conflicts with social rhythms, the discrepancy is defined as social jetlag (65). The anterior cingulate cortex (ACC), which is considered as a central hub in mood regulation, is a brain structure that shows the expression of biological clock genes (66). Social jet lag can influence emotional regulation by affecting the expression of circadian clock genes (67). Moreover, compared to morning-type individuals, night-type individuals may prefer to use mobile phones before bedtime. The blue-light emitted by mobile phones suppresses melatonin levels, which leading to circadian rhythm disruption (68), including social jetlag, and consequently intensifies the risk of negative emotions (69) and mental health disorders (70). To sum up, chronotype can regulate depression induced by mobile phone addiction by influencing social jetlag.

### 4.4 Policy and prevention

Our study reveals insights on the association between NLEs and depression, and what can regulate this relationship, which provides several practical implications for reducing the risk of developing depression among medical college students. Medical college students face intense academic pressure, negative experiences during clinical internships, and uncertainty about future career development. Therefore, it is of great significance to foster positive mental health. According to our results, the key prevention could include early identification and intervention of mobile phone addiction, as well as accurate identification of chronotypes. Colleges can incorporate scales about individual chronotypes and mobile phone usage in freshmen psychological assessment questionnaire. For individuals with suspected problematic mobile phone use, close monitoring and timely intervention should be implemented, especially during periods when negative life events are more likely to occur (such as examination months and internship periods). In addition, students should be encouraged to expose in morning outdoor light and take regular exercise, which can help individuals regulate clock gene expression and advance chronotype, thus preventing negative emotions (32).

### 4.5 Strength and limitations

The advantages of this study include: considered different dimensions of negative life events and analyzed their impact on depression, as well as explored the potential mechanisms underlying the relationship between NLEs and depression among medical college students. However, several limitations of the study should be taken into account. First, the cross-sectional design limited its ability to ascertain the causal relationships between NLEs, mobile phone addiction and depression. The directionality remains to be explored. What’s more, the sample of medical college students in this study was only from a medical college in north China, which may not represent national medical undergraduates, limiting generalizability. Therefore, longitudinal studies should be conducted to investigate causality between variables, particularly the controversial relationship between mobile phone addiction and depression. Besides, future studies can include samples from different regions as well as clinical samples to verify the robustness of our study’s conclusions.

## 5 Conclusions

Our study posted and verified a moderated mediation model in the association between NLEs and depression among medical college students, with mobile phone addiction played a partial indirect role. Moreover, we found that chronotype moderated the effect in the relationship between mobile phone addiction and depression: compared to students with morning chronotype, evening chronotype students had a stronger correlation between mobile phone addiction and depression. These findings may help medical colleges to implement intervention programs to prevent students’ depression when they are facing negative life events.

## Data Availability

The raw data supporting the conclusions of this article will be made available by the authors, without undue reservation.

## References

[1] Zhang J Zheng S Hu Z . The effect of physical exercise on depression in college students: The chain mediating role of self-concept and social support. Front Psychol. (2022) 13:841160. doi: 10.3389/fpsyg.2022.841160 35651580 PMC9150849

[2] Auerbach RP Alonso J Axinn WG Cuijpers P Ebert DD Green JG . Mental disorders among college students in the World Health Organization World Mental Health Surveys. Psychol Med. (2016) 46:2955–70. doi: 10.1017/s0033291716001665 27484622 PMC5129654

[3] Li W Zhao Z Chen D Peng Y Lu Z . Prevalence and associated factors of depression and anxiety symptoms among college students: A systematic review and meta-analysis. J Child Psychol Psychiatry. (2022) 63:1222–30. doi: 10.1111/jcpp.13606 35297041

[4] Zhu L Gong L Hu K Niu J Wang S Yang P . Understanding the correlation between benevolent childhood experiences, negative life events and mental health: The role of health risk behaviors. J Affect Disord. (2025) 390:119722. doi: 10.1016/j.jad.2025.119722 40541830

[5] Visser PL Loess P Jeglic EL Hirsch JK . Hope as a moderator of negative life events and depressive symptoms in a diverse sample. Stress Health. (2013) 29:82–8. doi: 10.1002/smi.2433 22552998

[6] Liu Y Zhang N Bao G Huang Y Ji B Wu Y . Predictors of depressive symptoms in college students: A systematic review and meta-analysis of cohort studies. J Affect Disord. (2019) 244:196–208. doi: 10.1016/j.jad.2018.10.084 30352363

[7] Zhang S Yang R Cui Y Zhou Y Jiang L Xi J . Negative life events, inadequate mental health literacy, and emotional symptoms among Chinese college students: A school-based longitudinal prospective study. Int J Ment Health Syst. (2025) 19:14. doi: 10.1186/s13033-025-00672-y 40307830 PMC12042421

[8] Beiter R Nash R McCrady M Rhoades D Linscomb M Clarahan M . The prevalence and correlates of depression, anxiety, and stress in a sample of college students. J Affect Disord. (2015) 173:90–6. doi: 10.1016/j.jad.2014.10.054 25462401

[9] Inam QU Shaikh S Wahab S Ovais MH Ahad Memon UA Anwar Z . Psychological impact of COVID-19 pandemic and associated factors on college students. J Pak Med Assoc. (2022) 72:2014–8. doi: 10.47391/jpma.4321 36660991

[10] Karhina K Bøe T Hysing M Nilsen SA . Parental separation, negative life events and mental health problems in adolescence. BMC Public Health. (2023) 23:2364. doi: 10.1186/s12889-023-17307-x 38031009 PMC10685480

[11] Hyde J Mezulis A Abramson L . The ABCs of depression: Integrating affective, biological, and cognitive models to explain the emergence of the gender difference in depression. psychol Rev. (2008) 115:291–313. doi: 10.1037/0033-295x.115.2.291 18426291

[12] Chen G Wang Y Li M Jia K Liu C Zhang Y . Mediation role of coping styles on negative life events and subthreshold depression in medical students. Curr Psychol. (2024) 43:36064–72. doi: 10.1007/s12144-024-06988-130311153

[13] Zhang Z Cai Z Meng Q . Negative life events, sleep quality and depression in university students. Sci Rep. (2025) 15:21193. doi: 10.1038/s41598-025-08635-6 40595267 PMC12216533

[14] Jiang P Zhang Z Li S Yan Y . Negative life events and depression among Chinese university freshmen: A longitudinal moderated mediation model. Front Public Health. (2024) 12:10. doi: 10.3389/fpubh.2024.1480394 39606075 PMC11598919

[15] Billieux J Maurage P Lopez-Fernandez O Kuss DJ Griffiths MD . Can disordered mobile phone use be considered a behavioral addiction? An update on current evidence and a comprehensive model for future research. Curr Addict Rep. (2015) 2:156–62. doi: 10.1007/s40429-015-0054-y30311153

[16] Turner A . How many smartphones are in the world? (2025). Available online at: https://www.bankmycell.com/blog/how-many-phones-are-in-the-world (Accessed August 11, 2026).

[17] Wang Y Abdullah MNLY . A meta-analytic review of smartphone addiction prevalence in Chinese college students. Curr Psychol. (2026) 45:548. doi: 10.1007/s12144-026-09160-z30311153

[18] Lyu C Cao Z Jiao Z . Changes in Chinese college students' mobile phone addiction over recent decade: The perspective of cross-temporal meta-analysis. Heliyon. (2024) 10:e32327. doi: 10.1016/j.heliyon.2024.e32327 38947462 PMC11214489

[19] Dan L Chen J Wu D Yin H . The relationship between negative life events and problematic social networking use among Chinese high school students: The moderating role of physical activity. Curr Psychol. (2025) 45:13. doi: 10.1007/s12144-025-08775-y30311153

[20] Ji M Qi Y Tu H Wu S Wang X . The influence of negative events on adolescents' mobile phone addiction: The chain mediating role of personality traits and emotional regulation style. Front Psychiatry. (2025) 16. doi: 10.3389/fpsyt.2025.1530212 40123602 PMC11926157

[21] Feng Z Diao Y Ma H Liu M Long M Zhao S . Mobile phone addiction and depression among Chinese medical students: The mediating role of sleep quality and the moderating role of peer relationships. BMC Psychiatry. (2022) 22:10. doi: 10.1186/s12888-022-04183-9 35999533 PMC9396829

[22] Pan J Guo J Wu Y Zhao X . The influence of negative emotions on mobile phone addiction among Chinese college students: The mediating role of negative coping styles and the moderating role of gender. Psychol Res Behav Manage. (2025) 18:3–13. doi: 10.2147/prbm.s497255 39802958 PMC11720637

[23] Kardefelt-Winther D . A conceptual and methodological critique of internet addiction research: Towards a model of compensatory internet use. Comput Hum Behav. (2014) 31:351–4. doi: 10.1016/j.chb.2013.10.05942574925

[24] Mi X Li X . The relationship between mobile phone addiction and depression, anxiety among Chinese college students: The mediating role of friendship quality and the moderating effect of preference for solitude. Front Psychiatry. (2026) 17. doi: 10.3389/fpsyt.2026.1859953 42459282 PMC13368748

[25] Kaya F Dastan N Durar E . Smart phone usage, sleep quality and depression in university students. Int J Soc Psychiatr. (2021) 67:407–14. doi: 10.1177/0020764020960207 32969293

[26] Ren X He R Tan J Huang M Liu Z . Mediating role of sleep quality of medical students in higher vocational colleges between mobile phone addiction and anxiety and depression. Medicine. (2025) 104:1.

[27] Gull M Sravani B . Do screen time and social media use affect sleep patterns, psychological health, and academic performance among adolescents? Evidence from bibliometric analysis. Child Youth Serv Rev. (2024) 164:9. doi: 10.1016/j.childyouth.2024.10788642574925

[28] Uyar G Yildiran H . The association among circadian rhythm, circadian genes and chrononutrition, its effect on obesity: A review of current evidence. Biol Rhythm Res. (2022) 53:1821–47. doi: 10.1080/09291016.2022.204463137339054

[29] Adan A Archer S Hidalgo M Di Milia L Natale V Randler C . Circadian typology: A comprehensive review. Chronobiol Int. (2012) 29:1153–75. doi: 10.3109/07420528.2012.719971 23004349

[30] Wallinheimo A-S Evans SL . Mechanisms that link circadian preference to problematic smartphone and social media use in young adults. PloS One. (2025) 20:e0331961. doi: 10.1371/journal.pone.0331961 40938835 PMC12431251

[31] Li T Zhang D Qu Y Zhai S Xie Y Tao S . Association between trajectories of problematic mobile phone use and chronotype among Chinese college students. Addict Behav. (2022) 134:107398. doi: 10.1016/j.addbeh.2022.107398 35752086

[32] Zhao Y Liao J Huang Q . Role of chronotype in depression. World J Psychiatry. (2025) 15:109087. doi: 10.5498/wjp.v15.i10.109087 41112620 PMC12531929

[33] Seizer L Martínez-Albert E Löchner J . Nonlinear and symptom specific associations between chronotype and depression. Sci Rep. (2024) 14:28696. doi: 10.1038/s41598-024-79868-0 39562745 PMC11576981

[34] Wittmann M Dinich J Merrow M Roenneberg T . Social jetlag: Misalignment of biological and social time. Chronobiol Int. (2006) 23:497–509. doi: 10.1080/07420520500545979 16687322

[35] Fabbian F Zucchi B De Giorgi A Tiseo R Boari B Salmi R . Chronotype, gender and general health. Chronobiol Int. (2016) 33:863–82. doi: 10.1080/07420528.2016.1176927 27148626

[36] Luo J Yang J Dai T Zhao J Wu S Zhou L . Non-linear relationship between social jetlag and depressive symptoms among Chinese college students. Sci Rep. (2025) 15:18317. doi: 10.1038/s41598-025-03371-3 40419605 PMC12106608

[37] Gao L Xie Y Jia C Wang W . Prevalence of depression among Chinese university students: A systematic review and meta-analysis. Sci Rep. (2020) 10:11. doi: 10.1038/s41598-020-72998-1 32985593 PMC7522998

[38] Pourhoseingholi MA Vahedi M Rahimzadeh M . Sample size calculation in medical studies. Gastroenterol Hepatol Bed Bench. (2013) 6:14–7. PMC4017493 24834239

[39] Taber K . The use of Cronbach's alpha when developing and reporting research instruments in science education. Res Sci Educ. (2018) 48:1273–96. doi: 10.1007/s11165-016-9602-230311153

[40] Schmitt N . Uses and abuses of coefficient alpha. psychol Assess. (1996) 8:350–3. doi: 10.1037/1040-3590.8.4.35027371692

[41] Kroenke K Spitzer R Williams J . The PHQ-9 - Validity of a brief depression severity measure. J Gen Internal Med. (2001) 16:606–13. doi: 10.1046/j.1525-1497.2001.016009606.x PMC1495268 11556941

[42] Zhang YL Liang W Chen ZM Zhang HM Zhang JH Weng XQ . Validity and reliability of Patient Health Questionnaire-9 and Patient Health Questionnaire-2 to screen for depression among college students in China. Asia Pac Psychiatry. (2013) 5:268–75. doi: 10.1111/appy.12103 24123859

[43] Adan A Almirall H . Horne & Östberg morningness-eveningness questionnaire: A reduced scale. Pers Individ Dif. (1991) 12:241–53. doi: 10.1016/0191-8869(91)90110-w

[44] Li W-X MuyeSeaizezi Zhi-Tao X Wu-Han L Bin Z University SM . Validity and reliability of the Chinese version of Morningness/Eveningness Questionnaire-5 items(MEQ-5) in students of technical schools. Chin Ment Health J. (2016) 30(6):406–12. doi: 10.3969/j.issn.1000-6729.2016.06.002

[45] Department of Medical Administration NHCotPsRoC . Guideline for diagnosis and treatment of obesity (2024 edition). Chin J Digestive Surg. (2024) 23:1237–60.

[46] Bolin J . Introduction to mediation, moderation, and conditional process analysis: A regression-based approach. J Educ Measurement. (2014) 51:335–7. doi: 10.1111/jedm.12050

[47] Wang Y Liang L Sun Z Liu R Wei Y Qi S . Factor structure of the patient health questionnaire-9 and measurement invariance across gender and age among Chinese university students. Medicine. (2023) 102(1):e32590. doi: 10.1097/md.0000000000032590 36607886 PMC9829284

[48] Wen L Zhang L Song J Feng Y Tao Y Li H . Depression and school adjustment among Chinese medical college students: The mediating role of loneliness and the moderating role of family functioning. Curr Psychol. (2025) 44:15472–83. doi: 10.1007/s12144-025-08297-730311153

[49] Liu D Zhang B . Survey and analysis of medical students' employment status in new era. Employment Guarantee. (2025), 84–6.

[50] Zhan H Zheng C Zhang X Yang M Zhang L Jia X . Chinese college students' stress and anxiety levels under COVID-19. Front Psychiatry. (2021) 12. doi: 10.3389/fpsyt.2021.615390 34177635 PMC8222572

[51] Yuanhua S Jing Y . The relationship between college students' depression and social inhibition. J Innovation Entrepreneurship Educ. (2025) 16:88–94.

[52] Zhou Y Chen S Zhang Y Yang Y Guo C . Socially prescribed perfectionism and depression: Roles of academic pressure and hope. School Ment Health. (2024) 16:518–29. doi: 10.1007/s12310-024-09655-930311153

[53] Gawai J Kasturkar P Uke T . Detecting the cause of depression using BECK depression scale among patients. J Pharm Res Int. (2021) 33:176–85. doi: 10.9734/jpri/2021/v33i35a3188735024509

[54] Carpar K Durmaz P Terler M . Evaluation of anxiety, stress, depression and hopelessness levels in clinical dental students of the faculty of dentistry: A cross-sectional study. BMC Med Educ. (2025) 26:10. doi: 10.1186/s12909-025-08530-8 41469987 PMC12860093

[55] Liu C Zhao Y Tian X Zou G Li P . Negative life events and school adjustment among Chinese nursing students: The mediating role of psychological capital. Nurse Educ Today. (2015) 35:754–9. doi: 10.1016/j.nedt.2015.02.002 25702847

[56] Wang M Ouyang L Xu Y Mi W Wang W Zhou L . The impact of negative life events on anxiety and depression among nursing freshmen: The mediating role of mobile phone addiction. J Psychiatr Ment Health Nurs. (2026) 33:174–83. doi: 10.1111/jpm.70078 41351354

[57] G L B G J LJ . An analysis of factors influencing the development of depressive symptoms among postgraduate students: The roles of adaptation challenges, employment stress, and romantic troubles. J Army Med Univ. (2026) 48:242–50. doi: 10.16016/j.2097-0927.202508044

[58] Higgins E . Self-discrepancy - a theory relating self and affect. psychol Rev. (1987) 94:319–40. doi: 10.1037/0033-295x.94.3.319 3615707

[59] Chen R Peng K Liu J Wilson A Wang Y Wilkinon M . Interpersonal trauma and risk of depression among adolescents: The mediating and moderating effect of interpersonal relationship and physical exercise. Front Psychiatry. (2020) 11. doi: 10.3389/fpsyt.2020.00194 32351408 PMC7174748

[60] Maajeeny H . Effects of family disintegration on children later depression. Int J Adv Appl Sci. (2022) 9:65–70. doi: 10.21833/ijaas.2022.03.008

[61] Yang Y Luo Y Chen M Zhai J . Life events, boredom proneness and mobile phone addiction tendency: A longitudinal mediation analysis based on latent growth modeling (LGM). Psychol Res Behav Manage. (2023) 16:2407–16. doi: 10.2147/prbm.s416183 37426385 PMC10327903

[62] Lin H Chang Y Chen M Liu S Chen B Li L . Structural and functional neural correlates in individuals with excessive smartphone use: A systematic review and meta-analysis. Int J Environ Res Public Health. (2022) 19:15. doi: 10.3390/ijerph192316277 36498362 PMC9739413

[63] Hafeman D . Proportion explained": A causal interpretation for standard measures of indirect effect? Am J Epidemiol. (2009) 170:1443–8. doi: 10.1093/aje/kwp283 19850625

[64] Roenneberg T Pilz LK Zerbini G Winnebeck EC . Chronotype and social jetlag: A (self-) critical review. Biology. (2019) 8:54. doi: 10.3390/biology8030054 31336976 PMC6784249

[65] Miño C Smith L Cristi-Montero C Gutiérrez-Espinoza H Olivares-Arancibia J Yañéz-Sepúlveda R . The hidden clock: How chronotype is related to depression, anxiety, and stress in adolescents – insights from the EHDLA study. Int J Ment Health Syst. (2025) 19:16. doi: 10.1186/s13033-025-00673-x PMC12096745 40405217

[66] Mendoza J Vanotti G . Circadian neurogenetics of mood disorders. Cell Tissue Res. (2019) 377:81–94. doi: 10.1007/s00441-019-03033-7 31073908

[67] Takahashi M Tahara Y Tsubosaka M Fukazawa M Ozaki M Iwakami T . Chronotype and social jetlag influence human circadian clock gene expression. Sci Rep. (2018) 8:10152. doi: 10.1038/s41598-018-28616-2 29976939 PMC6033857

[68] Li T Chan N Li C Chen J Chan J Liu Y . The associations of electronic media use with sleep and circadian problems, social, emotional and behavioral difficulties in adolescents. Front Psychiatry. (2022) 13. doi: 10.3389/fpsyt.2022.892583 35757219 PMC9218337

[69] Yue L Cui N Jiang L Cui N . Screen use before sleep and emotional problems among adolescents: Preliminary evidence of mediating effect of chronotype and social jetlag. J Affect Disord. (2023) 328:175–82. doi: 10.1016/j.jad.2023.02.049 36806592

[70] Walker WH Walton JC DeVries AC Nelson RJ . Circadian rhythm disruption and mental health. Transl Psychiatry. (2020) 10:28. doi: 10.1038/s41398-020-0694-0 32066704 PMC7026420

